# Sensorimotor Integration by Corticospinal System

**DOI:** 10.3389/fnana.2016.00024

**Published:** 2016-03-09

**Authors:** Yunuen Moreno-López, Rafael Olivares-Moreno, Matilde Cordero-Erausquin, Gerardo Rojas-Piloni

**Affiliations:** ^1^Departamento de Neurobiología del Desarrollo y Neurofisiología, Instituto de Neurobiología, Universidad Nacional Autónoma de México, Campus UNAM-JuriquillaQuerétaro, México; ^2^Unité Propre de Recherche 3212, Institut des Neurosciences Cellulaires et Intégratives, UPR 3212 CNRSStrasbourg, France

**Keywords:** corticospinal tract, spinal cord, dorsal horn, corticomotoneuronal, sensorimotor cortex, presynaptic inhibition

## Abstract

The corticospinal (CS) tract is a complex system which targets several areas of the spinal cord. In particular, the CS descending projection plays a major role in motor command, which results from direct and indirect control of spinal cord pre-motor interneurons as well as motoneurons. But in addition, this system is also involved in a selective and complex modulation of sensory feedback. Despite recent evidence confirms that CS projections drive distinct segmental neural circuits that are part of the sensory and pre-motor pathways, little is known about the spinal networks engaged by the corticospinal tract (CST), the organization of CS projections, the intracortical microcircuitry, and the synaptic interactions in the sensorimotor cortex (SMC) that may encode different cortical outputs to the spinal cord. Here is stressed the importance of integrated approaches for the study of sensorimotor function of CS system, in order to understand the functional compartmentalization and hierarchical organization of layer 5 output neurons, who are key elements for motor control and hence, of behavior.

## The Corticospinal Tract and the Sensorimotor Control

The sensorimotor cortex (SMC) has improved the capacity of mammals to learn and execute new and skillful movements (Nudo and Frost, 2009). Additionally, the SMC has access to contextual, sensory and planning information from other neocortical areas that may not be independently available to subcortical and spinal motor circuits. The SMC recruits phylogenetically older neuronal circuits in the magnocellular red nucleus, reticular formation and spinal cord to execute movements properly. In particular, the corticospinal tract (CST) is a key element of the motor command. The majority of the corticospinal (CS) connections with the motoneurons are established through premotor neurons (Lemon and Griffiths, 2005; Lemon, 2008), however, the direct connections of CST to motoneurons are the basis for hand dexterity such as the potential to control fractioned digit movements (Isa et al., 2013).

Additionally, any movement produces activation of peripheral receptors (proprioceptive and cutaneous) generating self-induced activity that is transmitted to the central nervous system where it interacts with motor commands and other processes (Rudomin, 1999; Rudomin and Schmidt, 1999; Seki et al., 2003). When entering the spinal cord, primary afferents can be modulated by primary afferent depolarization (PAD) leading to presynaptic inhibition of neurotransmitter release and hence modifying the inputs to second-order neurons (Hochman et al., 2010).

PAD, and hence presynaptic inhibition of different cutaneous and proprioceptive classes of sensory afferents, can be produced by stimulation of other sensory afferents (sensory feedback), but also by supraspinal structures, like the SMC (Carpenter et al., 1963; Andersen et al., 1964; Abdelmoumene et al., 1970; Aggelopoulos et al., 2008; Moreno-López et al., 2013) and pyramidal tract (Rudomin et al., 1986). This cortical control of sensory inputs in the spinal cord occurs in a very selective manner (Eguibar et al., 1994, 1997; Lomeli et al., 1998), which is crucial for motor control and for the proper execution of movements (Lebedev and Nelson, 1996). Moreover, presynaptic inhibition is important during voluntary movements (Hultborn et al., 1987a,b), and a descending system like the CST has a dominant role in producing presynaptic inhibition of sensory afferents compared to peripheral feedback resulting from movement. Furthermore, descending systems reduce specific peripheral inputs that can interfere with ascending commands and with spinal circuits during voluntary movement (Seki et al., 2003). This implies that the cerebral cortex could recruit spinal cord neuronal circuits that select the sensory information which is suitable for proper execution of a volitional movement, which is an additional level of sensorimotor integration.

## Spinal Cord interneurons Under Cortical Control

Spinal motoneurons are directly contacted by CS neurons in most primates, which is particularly important for high precision, manipulatory skills involving independent finger movements. However, an indirect cortico-motoneuronal connection through premotor interneurons has been also observed in both primates (Lemon, 2008) and in the mouse (Alstermark et al., 2004). Although, convergence from the CST and sensory afferents on segmental and propriospinal neurons projecting to motoneurons has been studied mainly in cats and primates, cellular and molecular properties of these premotor interneurons has been studied with more detail in rodents (Zagoraiou et al., 2009; Ni et al., 2014).

CST directly modulates segmental interneurons involved in sensory feedback, such that the interneurons responsible for PAD are directly activated by cortex stimulation (Carpenter et al., 1963). Moreover, a set of dorsal spinocerebellar tract (dSC) neurons, which receive proprioceptive-sensory inputs from group-I afferents also are activated by CST. Indeed the probability of dorsal root-evoked action potentials can be reduced by CS activity, and dSC neurons can also be directly inhibited by glycinergic and GABAergic inputs from interneurons activated by CS fibers. Thus, CST exerts presynaptic inhibitory control on a complex interneuronal system mediating the transmission from terminals of primary afferent fibers to spinocerebellar neurons (Hantman and Jessell, 2010).

More recently, it was shown that interneurons expressing the nuclear orphan receptor (RORα) participate in light-touch perception as well as in corrective foot movement and fine motor control (Bourane et al., 2015). RORα interneurons are also innervated by projection neurons of the lateral vestibular nucleus and CST. These interneurons integrate sensory inputs from cutaneous, low-threshold mechanoreceptors and descending motor signals from the cortex, suggesting an important implication of the CST in the modulation of sensory signals for the proper execution of volitive movements.

**Figure 1:**
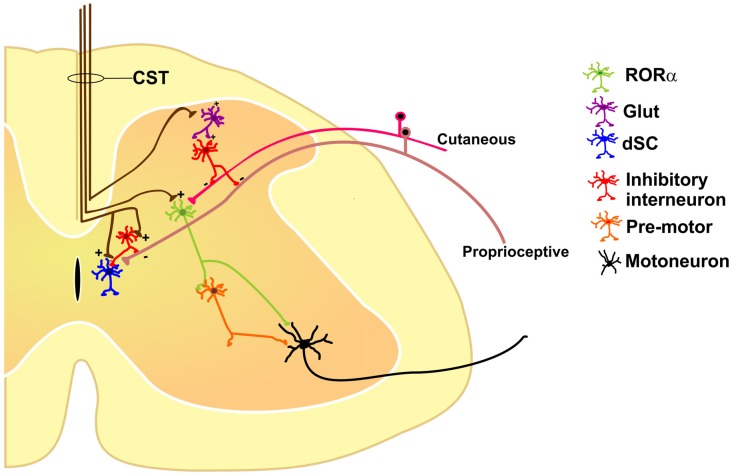
**Some of the identified segmental interneurons under corticospinal tract (CST) control are nuclear orphan receptor (RORα) (Bourane et al., 2015), dorsal spinocerebellar tract (dSC; Hantman and Jessell, 2010), and primary afferent depolarization (PAD)-mediated interneurons (Rudomin, 1999), all of which modulate sensory feedback.** See text for details.

All these results suggest that the cortex has diverse outputs by means of the CST, modulating different classes of excitatory and inhibitory spinal neurons and forming distinct spinal cord neuronal circuits which, together, participate in sensorimotor integration (Figure 1).

## Comparative Anatomy and Physiology of CST

The CST is present in all mammals and the general course of the CST is well-known in different species (Armand, 1982; Lemon, 2008). It originates from multiple motor and somatosensory cortices including premotor cortex, supplementary motor cortex, primary motor cortex, as well as primary and secondary somatosensory cortices (Ullan and Artieda, 1981; Miller, 1987; Galea and Darian-Smith, 1994; Lemon, 2008).

The CST provides the most direct pathway over which the cerebral cortex controls movement, using the primitive neuronal circuits into the spinal cord to generate motor output. CST axons terminate in all gray matter of the spinal cord (Armand, 1982), however in rodents and marsupials this influence is exerted largely upon interneurons in the dorsal horn of the spinal gray matter. Ascending the phylogenetic scale through carnivores and primates, the number of CS axons grows and CS terminations shift progressively toward the interneurons of the intermediate zone and ventral horn, ultimately forming increasing numbers of synaptic terminations directly on the motoneurons themselves in higher primates (for review, see Schieber, 2007).

CS neurons that make monosynaptic connections with motoneurons (corticomotoneuronal neurons) are a relatively new phylogenetic and ontogenetic development. Furthermore, in higher primates corticomotoneuronal cells are located in a separate part of the primary motor cortex than the CS cells contacting to spinal cord interneurons (Rathelot and Strick, 2009). These two CS cells are differentially involved in motor learning: CS cells contacting to spinal interneurons could be important during the initial stages of learning new skills by enabling the SMC to use existing spinal circuits to rapidly construct new movement patterns. In contrast, the new corticomotoneuronal connections may be especially important during the later stages of learning a new skill by enabling the motor cortex to refine and precisely specify patterns of motor output (Rathelot and Strick, 2009; Hudson et al., 2015).

It is self-evident that some characteristics features, such as skilled forelimb control associated with bipedalism, are product of specializations of CS system, like corticomotoneuronal connections from primary motor cortex. In contrast, other sensorimotor patterns have proven extremely successful, shaped by natural selection over extended periods of time. In this way, the CS descending control of sensory inputs from somatosensory cortices to the dorsal horn is found in all mammals (Lemon and Griffiths, 2005). But beyond the phylogenetic point of view, is clear that CST is a complex and diverse system with multiple targets within the spinal cord, in different laminae (dorsal/ventral) and/or at different segmental levels (Akintunde and Buxton, 1992; Biane et al., 2015; Kamiyama et al., 2015). In this way, sensorimotor integration mediated by CS system may has important consequences, especially considering that sensory modulation occurs within the dorsal spinal cord, and motor output triggered by premotor interneurons as well as motoneurons take place in the intermediate and ventral horn. Interestingly, in the upper cervical cord the projections of primary and secondary somatosensory cortex ramify extensively in the most dorsal and lateral regions of the spinal cord, while primary motor cortex projects more ventrally (Coulter and Jones, 1977; Suter and Shepherd, 2015).

It is tempting to propose that CST is functionally and structurally organized into different subsystems controlling in a coordinated manner distinct spinal cord circuits. This could be the case since layer 5 pyramidal neurons projecting subcortically are extremely diverse and heterogeneous class of cells. In addition to the spinal cord, they project to several structures like striatum, posteromedial thalamic nucleus, superior colliculus, pontine nuclei, red nucleus and reticular formation (Killackey et al., 1989; Akintunde and Buxton, 1992; Porter and Lemon, 1993; Hattox and Nelson, 2007; Groh et al., 2010). Moreover, all layer 5 neurons projecting to subcortical targets within the SMC form segregated populations projecting mainly individually to the subcortical structures (Jones and Wise, 1977; Akintunde and Buxton, 1992) and have electrophysiological (Hattox and Nelson, 2007) and morphological (Killackey et al., 1989) characteristics that distinguish each class. The impact of this differential projection remains to be established, although it could have functional relevance because all these targets are associated with different aspects of sensorimotor control.

## Future Directions to Uncover Corticospinal Functions

Sensory and motor functions are intimately related. Most movements give rise to an altered sensory environment (for example, proprioceptive, tactile, visual or olfactory); on the other hand, sensation is usually not passive but rather acquired in an active context (limb or eye movement, sniffing, etc.). Conceptually, the interaction between the sensory and motor systems should rely on feedback and closed-loop circuits. At the spinal cord level, the CST terminates not only in the ventral horns containing the motoneurons, but also massively in the dorsal aspects of the cord (Lemon, 2008), traditionally viewed as the “sensory” horn. This duality of function suggests that the cortex segregates these commands through different subpopulations of CS neurons, which may drive distinct populations of segmental inhibitory or excitatory interneurons like RORα, dSC and PAD-mediating interneurons or even other unexplored types (Figure 1).

**Figure 2:**
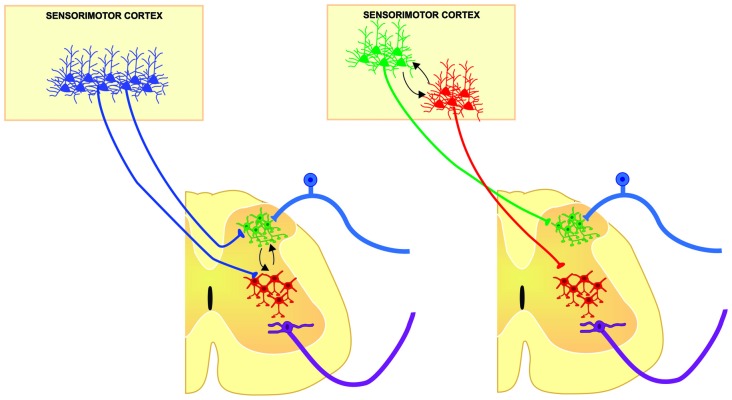
**Two main possibilities from which different populations of corticospinal (CS) neurons may modulate in a coordinated manner, distinct spinal cord neuronal circuits of the same segment, contributing to different aspects of sensorimotor integration.** Segmental interneurons involved in sensory modulation (green cells) could be modulated by the same (left) or different (right) population of CS neurons modulating the premotor circuits (red cells) in order to select the suitable sensory information and hence, increasing signal to noise ratio in motoneurons for proper execution of movements.

Historically, CST has been considered a unitary structure controlling motoneuron function. This view is now changing thanks to anatomical evidences showing the diversity of origin and CST connections in the spinal cord obtained in different species. Moreover, the fact that different populations of sensorimotor cortical neurons project in a segregated manner to different subcortical structures support the idea that also the CST is composed by subsystems controlling different spinal cord circuits that modulate motor outputs and sensory inputs in a coordinated manner. However, in order to fully know the physiological diversity of corticospinal system, experiments combining functional and anatomical approaches should be designed to disclose if segregated populations of corticospinal neurons modulates distinct spinal cord neuronal circuits. This could imply a functional compartmentalization and hierarchical organization between layer 5 output corticospinal neurons (Figure 2); therefore, future analysis of the organization of the cortical outputs to the spinal cord during movement would be important to understand the functional organization of the cortical circuits implicated in different aspects of motor control, and finally behavior.

## Author Contributions

YM-L and RO-M drafted the article. MC-E and GR-P edited the article. GR-P approved the final version.

## Funding

Supported by CONACYT (grant 176782) and PAPIIT (grant IN200615).

## Conflict of Interest Statement

The authors declare that the research was conducted in the absence of any commercial or financial relationships that could be construed as a potential conflict of interest. The reviewer RR and handling Editor declared their shared affiliation, and the handling Editor states that the process nevertheless met the standards of a fair and objective review.
